# Fluorescence Exclusion: A Simple Method to Assess Projected Surface, Volume and Morphology of Red Blood Cells Stored in Blood Bank

**DOI:** 10.3389/fmed.2018.00164

**Published:** 2018-05-30

**Authors:** Camille Roussel, Sylvain Monnier, Michael Dussiot, Elisabeth Farcy, Olivier Hermine, Caroline Le Van Kim, Yves Colin, Matthieu Piel, Pascal Amireault, Pierre A. Buffet

**Affiliations:** ^1^Biologie Intégrée du Globule Rouge UMR_S1134, Institut National de la Santé et de la Recherche Médicale, Université Paris Diderot, Sorbonne Paris Cité, Université de La Réunion, Université des Antilles, Paris, France; ^2^Institut National de la Transfusion Sanguine, Paris, France; ^3^Laboratoire d'Excellence GR-Ex, Paris, France; ^4^Université Paris Descartes, Paris, France; ^5^Laboratory of Cellular and Molecular Mechanisms of Hematological Disorders and Therapeutic Implications U1163, Centre National de la Recherche Scientifique ERL 8254, Institut National de la Santé et de la Recherche Médicale, Université Paris Descartes, Sorbonne Paris Cité, Paris, France; ^6^Institut Curie, Centre National de la Recherche Scientifique, UMR 144, PSL Research University, Paris, France; ^7^Assistance Publique des Hôpitaux de Paris, Paris, France; ^8^Institut Pierre-Gilles de Gennes, PSL Research University, Paris, France

**Keywords:** red blood cell volume, red blood cells, transfusion, red blood cell storage, fluorescence exclusion, red blood cell morphology

## Abstract

Red blood cells (RBC) ability to circulate is closely related to their surface area-to-volume ratio. A decrease in this ratio induces a decrease in RBC deformability that can lead to their retention and elimination in the spleen. We recently showed that a subpopulation of “small RBC” with reduced projected surface area accumulated upon storage in blood bank concentrates, but data on the volume of these altered RBC are lacking. So far, single cell measurement of RBC volume has remained a challenging task achieved by a few sophisticated methods some being subject to potential artifacts. We aimed to develop a reproducible and ergonomic method to assess simultaneously RBC volume and morphology at the single cell level. We adapted the fluorescence exclusion measurement of volume in nucleated cells to the measurement of RBC volume. This method requires no pre-treatment of the cell and can be performed in physiological or experimental buffer. In addition to RBC volume assessment, brightfield images enabling a precise definition of the morphology and the measurement of projected surface area can be generated simultaneously. We first verified that fluorescence exclusion is precise, reproducible and can quantify volume modifications following morphological changes induced by heating or incubation in non-physiological medium. We then used the method to characterize RBC stored for 42 days in SAG-M in blood bank conditions. Simultaneous determination of the volume, projected surface area and morphology allowed to evaluate the surface area-to-volume ratio of individual RBC upon storage. We observed a similar surface area-to-volume ratio in discocytes (D) and echinocytes I (EI), which decreased in EII (7%) and EIII (24%), sphero-echinocytes (SE; 41%) and spherocytes (S; 47%). If RBC dimensions determine indeed the ability of RBC to cross the spleen, these modifications are expected to induce the rapid splenic entrapment of the most morphologically altered RBC (EIII, SE, and S) and further support the hypothesis of a rapid clearance of the “small RBC” subpopulation by the spleen following transfusion.

## Introduction

Surface area-to-volume ratio is a major determinant of red blood cell (RBC) deformability and ability to circulate (1). Normal discocytes (8 μm in diameter) must indeed withstand stringent deformation as they navigate along 4- to 6-μm-wide microvessels and across 1- to 2-μm-wide inter-endothelial slits in the spleen. Modifications of surface area-to-volume ratio have consequences in physiology (removal of senescent RBC) (2, 3) and pathology (anemia in hereditary spherocytosis and other RBC hemoglobin and membrane disorders) (4–6). Furthermore, RBC display a tight relationship between morphology and deformability, and these parameters may impact transfusion yield. Previous studies have shown that RBC stored in blood bank exhibit morphological alterations that become significant after 3 or 4 weeks of storage depending on the technique and the cell classification (7–10). It has been also suggested that these alterations are associated with surface and volume modifications (11). A decrease in surface area-to-volume ratio induces a decrease in RBC deformability that can lead to their retention and elimination in the spleen (12–14). We recently showed that alterations in the morphology of stored RBC was accompanied by a decrease in the projected surface area that only impacts a subpopulation of RBC (10). The proportion of this subpopulation increases upon storage but is highly variable between donors. Determination of the projected surface area of these stored RBC was conducted using imaging flow cytometry, a high throughput tool allowing a detailed and objective quantification of cell morphology and dimensions (15). Imaging flow cytometry however cannot determine cell volume hence misses the key pathophysiological feature of RBC: surface area-to-volume ratio.

So far, single cell measurement of RBC volume has remained a challenging task achieved by a few sophisticated methods, such as micropipette aspiration (16, 17), quantitative phase microscopy (18) including digital holographic microscopy (19), some of these methods are flawed by potential artifacts due to labeling (confocal laser scanning microscopy) (20) or sphering (optical scattering methods) (21). A reproducible and ergonomic method enabling the assessment of the volume and morphology of RBC at the single cell level would be a useful tool to study the storage lesion and more generally RBC physiology and pathology.

The method developed here is based on the dye exclusion principle first proposed by Gray et al. (22) and adapted recently to mammalian cells (23–25). RBC were suspended in a medium supplemented with a fluorescent dye coupled to a dextran molecule and inserted in a microfluidic chamber of fixed height. Dextran is a biocompatible polysaccharide that does not cross cell membranes; fluorescence is thus excluded from the cells which allows volume calculation as the drop in fluorescence intensity is directly related to the thickness of the object (22). RBC volume is obtained by integrating the fluorescence signal over its projected area (see Material and Methods).

This technique also generates brightfield images enabling a simultaneous precise determination of their morphology.

## Materials and methods

### Chamber design and fabrication

Molds were fabricated using classic soft lithography methods or micromachining (Minimill3, Minitech Machinery) (24). Pillars were evenly positioned (interpillar distance 200 μm) in the observation chamber to set a very stable height in the chamber. Their value of fluorescence provides a stable signal useful for calibration (see Volume and projected area calculation in Material and Methods). Chips were made using a mixture 1:10 of PDMS (polydimethylsiloxane) and its cross linker (Sylgard 184, Dow Corning) cured at 66°C for 2 h. Inlets and outlets were created with 2 or 3 mm punchers before bonding. Chambers were bond on glass-bottomed petri dishes (Fluorodish) using air (Harrick) plasma cleaner or corona SB (Elveflow). Chamber surface was passivated with Poly(L-lysine) grafted poly(ethylene glycol) (PLL-g-PEG, Surface Solutions) for 30 min to 1 h after bonding. This pre-treatment induces the formation of a polymeric brush that prevents RBC from adhering to the surface. Chambers can be used immediately or stored at 4°C in PBS solution for a few days before use. Prior to RBC injection, PBS was changed to Krebs-Albumin 0.5% solution (Krebs-Henseleit Buffer modified with 2 g glucose, 2.1 g sodium bicarbonate, 0.175 g calcium chloride dehydrate and 5 g AlbuMAX II Lipid-Rich BSA for 1 L sterile water, pH 7.4) supplemented with FITC-dextran. According to its composition, Krebs-Albumin medium should have a refractive index similar to classical cell culture medium and thus be estimated at 1.337 (26).

### Sample preparations

Leukoreduced RBC in Saline-Adenine-Glucose-Mannitol (SAG-M) from healthy donors were supplied by the Etablissement Français du Sang (French Blood Service) 3 days after blood collection. All units were stored in optimal blood bank conditions between 2 and 6°C and for 42 days, according to regulations. Samples were aseptically collected to perform experiments. Just before analyses, RBC were diluted (1/50) in a Krebs-Albumin 0.5% solution. pH-related morphological alterations were induced by suspension of RBC in a Krebs-Albumin 0.5% solution after adding either HCl or NaOH up to the desired pH. Heated-RBC (HRBC) were produced by incubation of a RBC suspension at 1% hematocrit in RPMI at 50°C in a glass tube for 20 min. We used HRBC and RBC exposed to low and high pH as well-known examples of clear-cut volume and surface modifications RBC. In addition, HRBC have been repeatedly used to measure the biomechanical retention of stiff RBC *in vivo*, including in human subject (27, 28).

### Fluorescence exclusion sample preparation

RBC were suspended in the medium supplemented with 1 mg/mL FITC-Dextran (10 kDa) from Sigma-Aldrich. The hydrodynamic radius of the FITC-Dextran used in this study has been determined previously to be 1.86 nm (29) and thus far exceeded the maximum pore radius of the erythrocytes membrane (0.4 nm) (22, 30). RBC concentration was adjusted by modifying the dilution from 1:30 to 1:50 in order to obtain an optimal number of RBC in the chamber and avoid superimposition of RBC or contacts between them that would hamper measurements.

### Imaging

Imaging was performed with an automated Nikon Eclipse-Ti microscope equipped with a 20x objective NA.0.75, or a Zeiss AxioObserver microscope equipped with a 20x objective NA.0.8. Compatibility and precision of such objectives have already been shown (25). Fluorescence and brightfield images were acquired sequentially within <0.5 s according to the microscopes manufacturers.

### Volume and projected area calculation

Volume calculation was performed as described (24). Briefly, image analysis was performed using homemade MatLab program (The Math Works Inc., Natick, MA, USA). Calibration of the relationship between fluorescence intensity and height was performed for each field using values of fluorescence around each RBC and over the pillars as described (25). Briefly, α is extracted using a robust fit from *I*_*B*_ = α · *h* + *I*_0_, where *I*_*B*_ and *I*_0_ are the background and pillar fluorescence respectively and *h* the height of the chamber. To correct for the inhomogeneity of the fluorescent lamp, background is locally subtracted and the volume is then calculated by integrating over an area *S* larger than cell ($V_{Cell}=\iint_{S}\frac{I_{B}\left(x,y\right)-I\left(x,y\right)}{\alpha }dS$). This procedure also integrates the dye deposition on the chamber walls that can occur after several hours. After background removal, the fluorescence value around the RBC is close to zero, the area of integration does not play a crucial role and thus limits errors that could be introduced by a precise segmentation of the cell area. Projected area was extracted from brightfield images after background removal by a basic thresholding and filling procedure using built-in MatLab functions. Briefly, a square region of interest (ROI) was defined around the cell, then a binary gradient image was obtained using the edge function, the outlines of the binary image were dilated (imdilate) and the open area were filled (imfill). Eventually, objects too small or on the edge of the ROI were discarded (imclearborder, imerode). Parameters used during this procedure were adapted by user accordingly to images quality and exposure properties.

### Morphological analysis of RBC

After acquisition, brightfield images where anonymized and randomized for blind evaluation and RBC were classified in 6 morphological categories according to Bessis et al. (31). adapted for DIC microscopy (10). namely discocytes, echinocytes (I, II and III), sphero-echinocytes and spherocytes. Small RBC were defined as previously described (10) as a morphologically altered RBC population that exhibited a decrease in projected surface area and included 3 subpopulations, namely echinocytes III, sphero-echinocytes and spherocytes.

## Results

### Determination of RBC volume by fluorescence exclusion is simple and requires limited specific instruments

**Figure 1:**
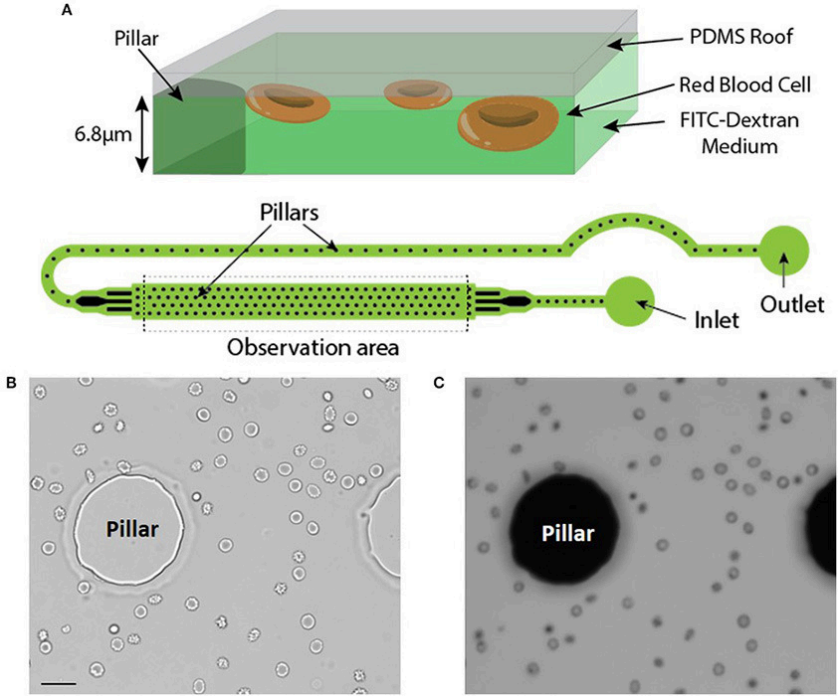
**(A)** Top: Principle of the fluorescence exclusion method, bottom: design of the microfluidic chamber. **(B,C)** Brightfield and fluorescence images of the same field (x20) showing RBC stored for 42 days in SAG-M (healthy donor) scale bar = 20 μm.

RBC were injected in a microfluidic chamber higher than the maximum size of the cells to prevent mechanical stress (chamber height = 6.8 μm). Its height was however limited to enable the capture of images with excellent contrast. The microfluidic chamber was designed to determine the volume of large numbers of RBC within a single chamber (typically from 500 up to 1,500 RBC depending on the working concentration). Regularly spaced pillars (interpillar distance 200 μm) ensure a constant height across the whole chamber and enable the calibration of the relationship between height and fluorescence (see Material and Methods). The inlet and the outlet were in immediate proximity and a fluid bridge was established between them to easily stop the flow. This prevented any fluid flow circulation in the chamber thereby enabling measurements on still RBC (Figure 1A).

Brightfield (Figure 1B) and fluorescence (Figure 1C) images were acquired using a 20x objective and each acquired fluorescence image enabled the volume determination of 10 to 50 RBC, depending on the concentration. Determination of RBC morphology can be evaluated simultaneously by acquiring and analyzing the corresponding brightfield image and was achieved according to Bessis et al. (see Methods).

**Figure 2:**
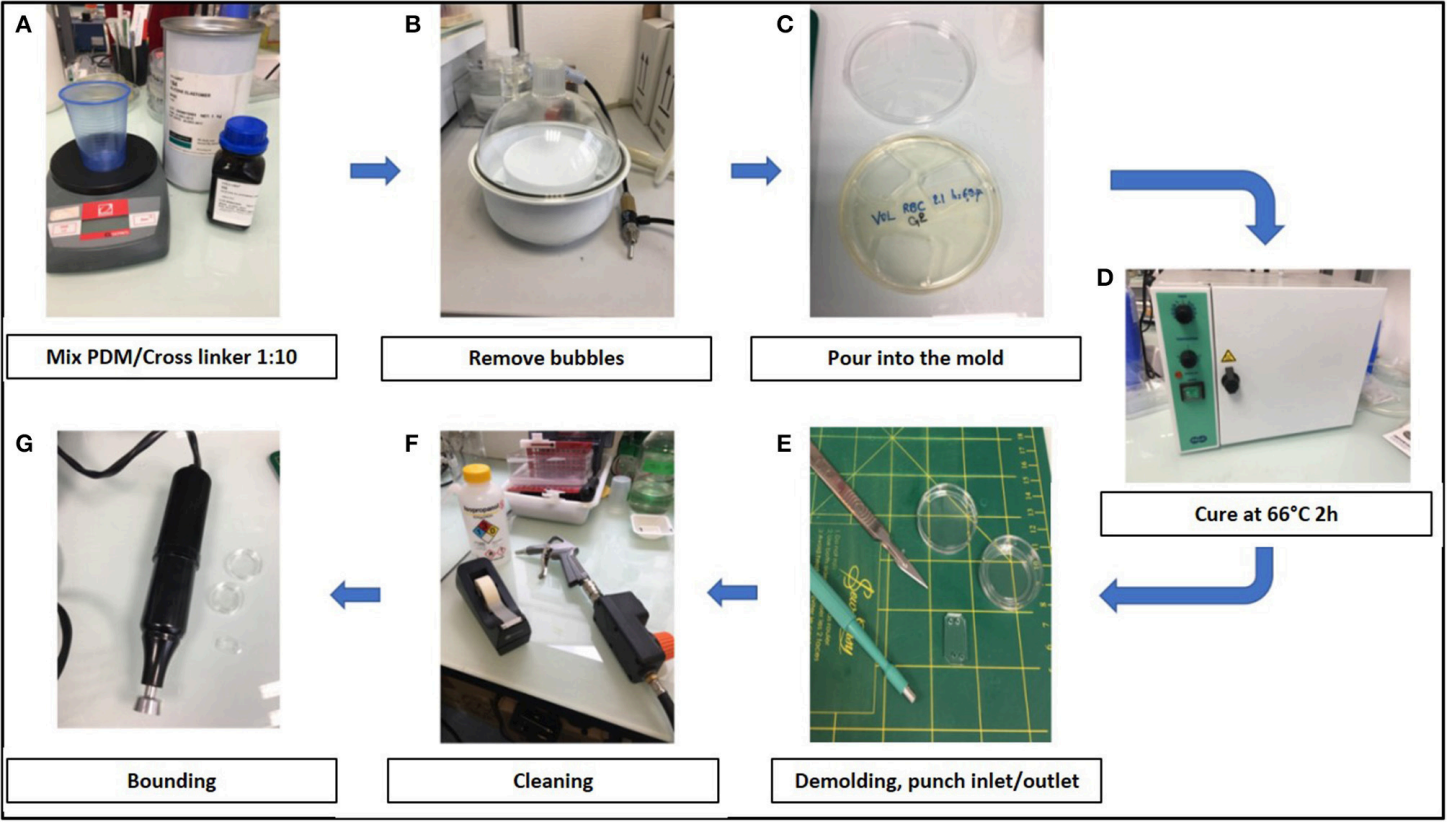
Stepwise preparation of chips: **(A)** Chips were made using a mixture 1:10 of PDMS (polydimethylsiloxane) and its cross linker (Sylgard 184) **(B)** The mixture was degased to remove air bubbles, **(C)** poured into the mold and **(D)** cured at 66°C for 2 h. **(E)** After demolding, inlets and outlets were created with 2 or 3 mm punchers and **(F)** the surface was cleaned using isopropanol, air gun and tape before bonding. **(G)** Chambers were bond on glass-bottomed petri dishes (Fluorodish) using air plasma cleaner (Harrick) or a corona SB (Elveflow).

Training to prepare chips required a few hours to a few days. The air plasma cleaner was replaced by a cheaper corona SB (Elveflow) that efficiently activated the surfaces before bonding (see Methods). Manufacturing steps are shown in Figure 2.

### The RBC volume quantification by fluorescence exclusion is precise and reproducible

**Figure 3:**
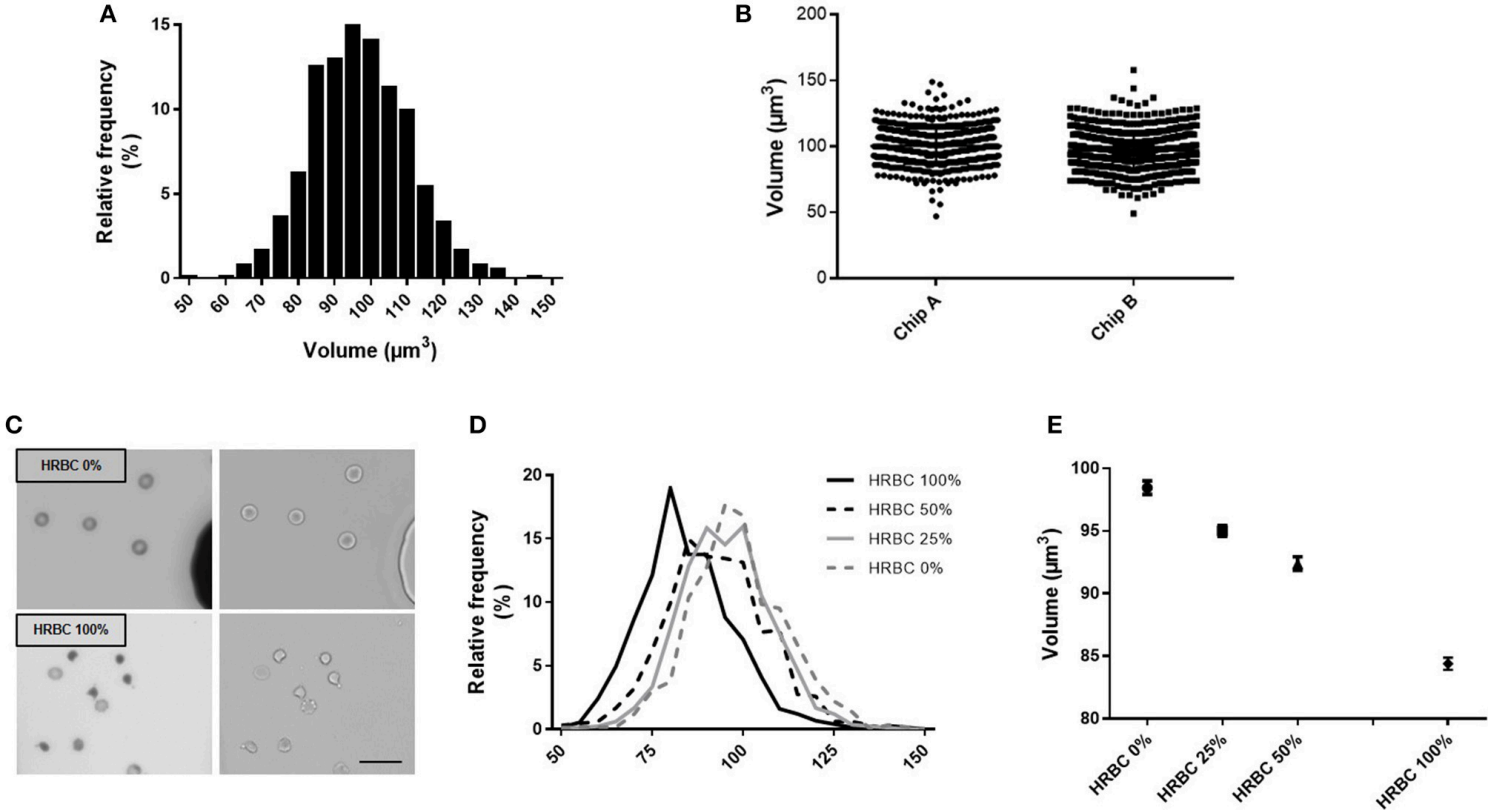
Fluorescence exclusion to measure RBC volume: a precise and reproducible method. **(A)** Normalized frequency histogram of RBC volume distribution from a healthy donor at day 10 of storage in SAG-M. **(B)** Individual volume of RBC from a healthy donor measured using 2 differents chips manufactured from the same mold (chip A and chip B). **(C)** Fluorescence (left) and brightfield (right) images (20x) of control RBC (top) or 100% heated RBC (bottom) scale bar = 20 μm; **(D)** Normalized frequency histogram of RBC volume distribution, and (E) Mean volume (+/−SEM) of samples containing increasing concentrations of heated-RBC.

To explore the reproducibility of the chip preparation and technique, the RBC volume from a RBC concentrate at day 10 of storage was assessed, on the same day, in two chips (namely A and B), manufactured from the same mold. Volume measurement showed a normal gaussian distribution (Figure 3A). Comparison of RBC volume between the two chips revealed a difference in the median volume of 3% (97 μm^3^ in chip A vs 100 μm^3^ in chip B) and a similar distribution (Figure 3B). The repetition of this experiment showed a variation of the median volume of 1.6% (97.4 vs. 99 μm^3^) (data not shown). This difference is very likely due to variations in chip height that originate from the master mold itself (one mold contains several chip designs for multiplexing the PDMS chip fabrication) as the error on the height is also 3%. Other sources of error would include, the chip bonding to glass, local variations from height variation within one chip. The background cleaning step during the image processing would also contribute, but only to width distribution and not to the inter chip variations.

Precision of the technique was next assessed using samples containing an increasing concentration of RBC of reduced volume (Figure 3C). HRBC were used since they are known to display a loss of volume. Normalized frequency histogram of RBC volume distribution showed a progressive shift to the left when the proportion of heated RBC increased in the sample (0, 25, 50, and 100%) (Figure 3D). Mean volume (± SEM) of each sample was 98.4 (±0.54) μm^3^, 95.0 (±0.44) μm^3^, 92.4 (±0.54) μm^3^, and 84.4 (±0.48) μm^3^ respectively (Figure 3E).

### Fluorescence exclusion measures volume while defining RBC morphology

**Figure 4:**
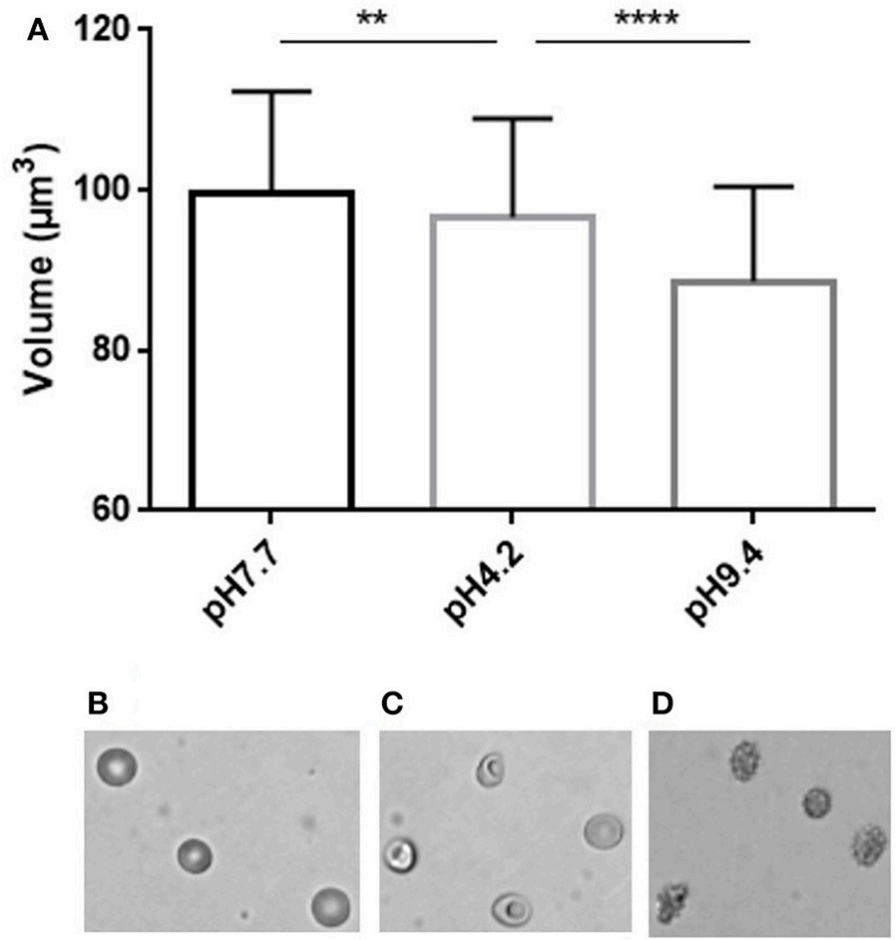
Simultaneous determination of RBC volume and morphology using fluorescence exclusion and microscopy. **(A)** Mean (standard deviation) volume of RBC samples exposed to physiological (left), low (center) and high (right) pH. Stars represent the significance level of the Mann-Whitney statistic test, ** when *p* < 0.01, **** when *p* < 0.0001 **(B–D)** Representative brightfield images of RBC in the same samples.

We next incubated RBC in media of acidic or basic pH to assess the RBC volume modifications associated with the pH-induced morphological modifications (Figure 4). Acidic pH (4.2) generated stomatocytes and sphero-stomatocytes (Figure 4B) which exhibited a mean 3% volume loss compared to physiological conditions (pH 7.4) (Figure 4C) while basic pH (9.4) generated echinocytes III, sphero-echinocytes and spherocytes (Figure 4D) that had lost a mean 11.4% of their volume. Differences in RBC volume were significantly different when measured either at low or normal pH (*p* < 0.01, ^**^ Mann Whitney non-parametric test) and between high and low pH (^****^p < 0.0001).

### Fluorescence exclusion shows a decrease in surface area-to-volume ratio in red blood cell stored in blood bank conditions

**Figure 5:**
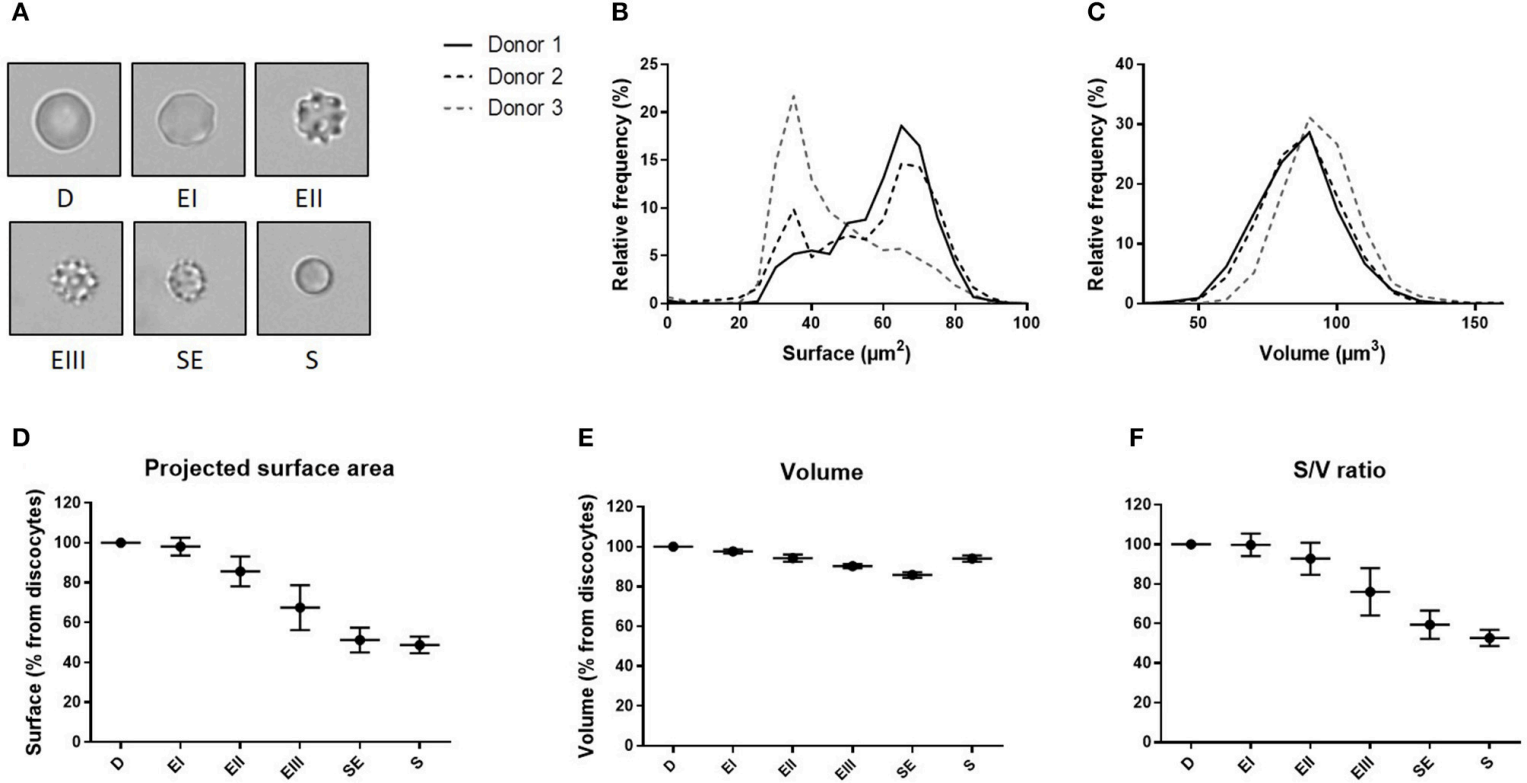
Fluorescence exclusion shows a decrease in surface to volume ratio during storage of red blood cell concentrates. **(A)** Morphological categories of RBC as defined in the Material and Methods section, namely discocytes (D), echinocytes I (EI), echinocytes II (EII), echinocytes III (EIII), spheroechinocytes (SE) and spherocytes (S). **(B–C)** Normalized frequency of RBC projected surface area (μm^2^) and RBC volume (μm^3^) of RBC concentrates after 42 days of storage in SAG-M (*n* = 3). **(D-F)** Mean (SD) projected surface area, volume and projected surface area-to-volume ratio normalized to the value of discocytes (D).

We determined the volume and the projected surface area of RBC stored for 42 days in 3 blood bank concentrates from healthy donors and correlated this measure with their storage-induced morphological alterations (Figure 5A). For the 3 donors, surface distribution on normalized frequency plots was bimodal and confirmed the existence of a subpopulation of “small RBC” with a reduced mean projected surface area (<58 μm^2^) (Figure 5B). Mean volume (±SD) was 86.0 ± 14.4 μm^3^ for donor 1, 85.2 ±14.1 μm^3^ for donor 2 and 93.9 ± 13.2 μm^3^ for donor 3 (Figure 5C). We then determined the projected surface area, volume and surface area-to-volume ratio of RBC for each morphological category (Figures 5D–F).

We observed a projected surface area loss of altered RBC when compared to discocytes (D): Echinocytes (E) I, II and III exhibited a mean projected surface area loss of 2, 14, and 32% respectively while the most intensely altered RBC, namely spheroechinocytes (SE) and spherocytes (S) had lost 49 and 51% of their projected surface area, respectively. Altered RBC exhibited also a decrease in their volume although to a lesser extent. When compared to D, the mean volume loss of storage-damaged RBC was 2% (EI), 6% (EII), 10% (EIII), 14% (SE) and 6% (S). This resulted in a surface area-to-volume ratio not modified for EI and decreased of 7 and 24% for EII and EIII respectively, and of 41 and 47% for SE and S. Small RBC (EIII, SE and S) exhibited a mean decrease in surface area and volume of 40 and 11% respectively resulting in a decrease of surface area-to-volume ratio of 32%.

## Discussion

We adapted the fluorescence exclusion method to determine RBC volume. This method is reproducible and sensitive and allows the simultaneous measurement of volume and projected surface area together with a detailed determination of RBC morphology at a single cell level.

Several methods such as automated complete blood count and impedance-based Coulter-Counter have been described to measure cell volume, but while of major clinical impact, they have disadvantages regarding mechanistic exploration of RBC. These high-throughput methods indeed provide reproducible data but volume can only be measured on global populations of RBC and not at a single cell level (32). Light scattering in flow cytometry is also a high-throughput method which is frequently used because of the wide availability of flow cytometers. It measures individual RBC volume together with hemoglobin concentration but requires pre-treatment of the cell (sphering). Furthermore, flow cytometry does not enable fine morphological observation of RBC (32–34). Morphology, volume and surface of single RBC can be accurately assessed by micropipette aspiration (16, 17) which also provides biomechanical information such as membrane viscosity and multiple elastic moduli. Micropipette measures are however of low throughput, technically challenging, and mastered by a very few specialized teams. Moreover, mechanical constraints on the RBC during micropipette aspiration may generate artifactual changes in morphology or volume. Lately, new techniques to obtain RBC volume have been proposed, based on sophisticated microscopical methods and microfluidics. Guo et al. have generated a microfluidic device to measure RBC volume based on electric current modification as cells pass through a gate (MOFSET-based detection) (35). Confocal microscopy can also be used but, in addition to requiring a specific instrumentation, this technique requires membrane labeling that can modify RBC morphology (19). Quantitative phase imaging, including digital holographic microscopy can provide RBC volume and RBC refractive index, but these two parameters are measured separately in isotonic liquids of different refractive indexes and thus requires RBC adhesion to the surface or complex microfluidic setup in order to perfuse solutions (19, 36). Scanning electron microscopy has also been proposed, but its usefulness is limited by a very low throughput and the limited availability of the instrument. Not least cell fixation is required and known to induce both morphological and volume changes.

The principle of dye exclusion has been recently adapted to RBC by Schonbrun et al. (26). RBC are suspended in an index-matching absorbing solution and volume can be measured from the modification in light absorbance between the cells and the background (37). Measures with this technique are independent from cell refractive index and provide microscopic spatial resolution of single cells as well as medium throughput, but microfluidic controllers are needed.

Unlike most aforementioned methods, the fluorescence exclusion technique described here does not require any pre-treatment of RBC and can be performed in physiological buffer. and could then be used for long term experiments with multiple cell types [see Cadart et al. (25)]. These 2 experimental features are real assets when studying RBC. Morphology and dimensions of RBC (especially those of altered RBC) are indeed exquisitely labile and sensitive to any fluctuation in the pH of the medium, its composition and most labeling procedures. Also, the chips are loaded without requiring pressure control or any specific infusion device. RBC adherence to the surface is not warranted since the immobility of the RBC is obtained by creating a bridge of fluid between the inlet and the outlet. Not least, measuring fluorescence exclusion only requires a fluorescence microscope.

We showed that fluorescence exclusion can detects variations of volume of a RBC population as small as 3%. Mean volume of RBC populations containing increasing proportions of small heated RBC were very close to theoretical predictions. RBC heated at 50°C during 20 min exhibited a 14% volume loss. Samples containing 25 and 50% heated-RBC should have exhibited 3.5 and 7% volume loss while measured values were 3.5 and 6.1%, respectively. Reproducibility was robust when measuring the mean RBC volume in the same samples using 2 different chips. The method still has weaknesses however. In its current version it displays a relatively low throughput. Improvement is envisioned by combining the analysis program to an algorithm for automated classification of RBC morphology, as described by Piety et al. (38). Also, like imaging flow cytometry, our method measures the projected surface area of RBC, a proxy for the total RBC surface generally considered accurate but that may be suboptimal for echinocytes that exhibit membrane spicules.

Using this new method, we confirmed and expanded recent findings. We measured the projected surface area, volume and surface area-to-volume ratio of RBC stored 42 days in blood bank conditions.

Measures of the volume of the subpopulation of “small RBC” (EIII, SE and S) by fluorescence exclusion provided direct confirmation that this subpopulation exhibits an overall volume loss of 11%. Because surface loss was proportionally greater than volume loss, the result was a reduced surface area-to-volume ratio of 32%. Previous observations had shown that a reduction in surface area-to-volume ratio was correlated to splenic retention of RBC. A reduction> 21% had led to a rapid entrapment of 79% of RBC in normal human spleens perfused *ex-vivo* (14). If RBC dimensions determines indeed the ability of RBC to cross the spleen, EIII, SE and S induced by storage (i.e small RBC), that exhibit a decrease in surface area-to-volume ratio of 24, 41, and 47% respectively (resulting in an overall decrease of 32%), are expected to undergo splenic retention. Not least, the marked difference in projected surface area-to-volume ratio observed between EII and EIII, as well as between EIII and SE is consistent with the similarly marked difference in the capacity of these RBC subsets to circulate in a microfluidic device (39). These data further support the hypothesis of a rapid clearance of “small RBC” by the spleen following transfusion. Recent publications showed that approaches consisting in reducing the appearance (anaerobic storage) or selectively remove this sub-population (washing in hypotonic solution) improved the ability of stored RBC to perfuse in an artificial microvascular network (40, 41).

Little is known about volume modification of stored RBC at the single cell level. Several studies showed an increase in RBC mean corpuscular volume (MCV) upon storage (42, 43) but a recent studies using quantitative phase imaging observed no significant volume modification after 6 weeks of storage (36, 44). We observed that the volume of RBC stored in SAG-M for 42 days was smaller in EI, EII, EIII, and SE than in D (with an almost linear decrease from EI to SE). The volume of S, although reduced, was greater than that of EIII and SE. In the context of transfusion, we found no previous direct observation on the volume of the different morphological subpopulations of RBC. We are thus currently unable to address the external consistency of this somewhat unexpected observation. In physiology, RBC with irregular shape in pre-term and term neonates show a similar decline in volume from D to SE but the volume of S is lower than that of SE, when assessed by micropipette aspiration (26). The higher-than-expected volume of S that we observed using fluorescence extinction may be artefactual or correspond to alterations of transmembrane flow of ions and water, as S are the most altered RBC subpopulation in red blood cell concentrates. This may have escaped observations with micropipettes because mechanical constraints linked to the aspiration may artificially modify RBC volume. When submitted to mechanical forces, RBC can indeed undergo dehydration via the activation of the mechanosensitive cation channel, Piezo 1 (45). By contrast, our method does not require manipulation of RBC and the height of the chips (6.8 μm) protects RBC from stringent mechanical forces. These hypotheses require experimental testing. Which method is the most accurate to quantify the volume of S will be determined by future work, for example by direct comparisons using all available methods with an array of altered RBC.

The method described here has the potential to bring important insight into the ability of RBC to circulate, in the context of RBC transfusion but also in RBC membrane or volume disorders.

## Author contributions

CR, SM, OH, CL, YC, MP, PA, and PB: designed the research; CR, SM, MD, and EF: performed the experiments; CR, SM, MD, and EF: analyzed the data; SM, CR, PA, and PB: wrote the paper.

### Conflict of interest statement

PA and PB have a sponsored research agreement with Zimmer Biomet. The other authors declare that the research was conducted in the absence of any commercial or financial relationships that could be construed as a potential conflict of interest.

## Abbreviations

D: Discocyte
E: Echinocytes
SE: spheroechinocytes
S: spherocytes
RBC: red blood cell
MCV: Mean Corpuscular Volume
SAG-M: Saline-Adenine-Glucose-Mannitol
CPDA: Citrate-Phosphate-Dextrose-Adenine
RPMI: Roswell Park Memorial Institute medium.
